# The relationship between sleep duration and frailty: findings from the China Health and Retirement Longitudinal Study

**DOI:** 10.3389/fpubh.2024.1493533

**Published:** 2024-12-23

**Authors:** Liyan Huang, Xiaofang He, Yao Zuo, Hui Yang, Lin Zhang

**Affiliations:** ^1^Department of Neurology, Xuanwu Hospital, Capital Medical University, Beijing, China; ^2^Department of Nursing, Guizhou Provincial People’s Hospital, Guiyang, China; ^3^Department of Neurology, Affiliated Hospital of Guizhou Medical University, Guiyang, China; ^4^Department of Neurology, The Second Affiliated Hospital of Guizhou University of Traditional Chinese Medicine, Guiyang, China

**Keywords:** sleep duration, frailty, frailty index, CHARLS, Chinese middle-aged and older adults

## Abstract

**Background:**

Research investigating the association between sleep duration and the risk of frailty has yielded conflicting results. This study used data from the China Health and Retirement Longitudinal Study (CHARLS) to investigate the association between sleep duration and frailty.

**Methods:**

Participants aged 45 and above at baseline were included in this study. Night or total sleep was categorized into three groups: short (<6 h), normal (6–9 h), and long sleep duration (≥9 h). Frailty was measured by a 31-item frailty index (FI). Non-frail participants at baseline were followed up after a 7-year period. The association between sleep duration and FI was examined by linear regression and restricted cubic spline (RCS) analysis. The relationship between sleep duration and the risk of frailty was evaluated using multinomial logistic regression analysis.

**Results:**

A total of 10,258/10,250 (night/total sleep duration) participants were included in the cross-sectional study and 4,770/4,768 in the longitudinal study. A negative correlation was identified between the both night and total sleep duration and FI (night: *β* = −0.83, *p* < 0.001; total: *β* = −0.66, p < 0.001) after adjusting for age, sex, education level, marital status, residence, yearly expenditure, BMI, waist circumference, smoking status, and drinking status in the cross-sectional study. In the longitudinal study, the relationship remained. Short sleep duration increased FI (night: *β* = 3.59, *p* < 0.001; total: *β* = 3.74, *p* < 0.001) and the risk of frailty (night: OR [95% confidence interval (CI)], 1.06 [1.05, 1.08], *p* < 0.001; total: 1.07 [1.06, 1.08], *p* < 0.001) compared to normal sleep duration in the fully adjusted model of the cross-sectional study. The result remains consistent in the longitudinal analysis.

**Conclusion:**

Short sleep duration increases the risk of frailty in Chinese individuals aged 45 and above. Extending sleep duration in this population may help prevent or alleviate frailty among middle-aged and older adult individuals.

## Introduction

The global aging population is projected to reach 2 billion people by the year 2050, with frailty emerging as one of the most significant challenges. Frailty is a significant clinical syndrome associated with aging, characterized by heightened susceptibility to stressors due to a progressive deterioration in various physiological systems (1). The gradual decline in homeostatic reserve may lead to a notable decline in functional abilities when encountering relatively minor stressors, such as acute infections or minor surgeries. In this case, the term “frailty” is used to describe the accelerated decline in homeostatic mechanisms, aiming to evaluate the vulnerability and prognosis of older adult individuals. Frailty prevalence varies from 10 to 37% among community residents and from 18 to 40% among hospitalized patients (2, 3). It is estimated to affect 25–50% of individuals aged over 85 years. Valid frailty models have identified the relationship between frailty and adverse health outcomes, such as falls, disability, restricted mobility, dependency, hospitalization, and mortality (4, 5). Given the significant medical and socioeconomic implications, it is imperative to identify older adult individuals at high risk for frailty and implement suitable interventions to prevent its development and advancement.

The two common tools for assessing frailty in both research and clinical settings are the Fried phenotype criteria and FI. The former comprises five specific symptoms: weakness, slowness, exhaustion, low physical activity, and shrinking. The latter was measured by the accumulation of deficits, including comorbidities, disabilities, ability in the activities of daily living, and so on (6). FI is a better predictor of adverse health outcomes than the frailty phenotype because it has a more finely graded risk scale and takes into account comorbidities and disabilities (7). Research has established that an index containing 30–40 variables can accurately predict adverse outcomes. Within this range, the results were usually consistent regardless of the number or specific terms of deficits used (8).

Given that frailty is not inherently irreversible, it is crucial to identify relevant risk factors to reduce its occurrence. Sleep is an essential component of daily life. Previous research has suggested a link between sleep duration and the development of frailty. However, the findings have been inconsistent. A correlation between both long (≥9 h) and short sleep duration (≤6 h or ≤ 5 h) and an increased frailty risk has been identified in Japan, the Netherlands, and Denmark (5, 9, 10). Conversely, some studies found frailty is only linked to long sleep duration in the United States, Korea and Greece (5, 11, 12). Nevertheless, other studies were unable to establish the above correlation (13, 14). The studies conducted in China were also controversial, and a clear general conclusion is lacking (5, 15–17). The majority of these studies used the Fried phenotype whereas the FI criterion was rarely assessed. Additionally, most previous studies were cross-sectional, which may introduce the possibility of reverse causation. Since sleep is a modifiable factor in the daily lives of the older adult, more evidence needs to be gathered before short or long sleep can be firmly established as a behavioral risk factor for frailty. This study used data from the CHARLS to investigate the relationship between short and long sleep duration and frailty.

## Methods

### Study population

The CHARLS, initiated in 2011, is a longitudinal, nationally representative survey conducted in China.^1^ The study investigated individuals aged 45 years and older together with their spouses in 150 counties/districts and 450 villages/urban communities across 28 provinces (including autonomous regions and municipalities) through a multistage stratified probability-proportional-to-size sampling method (18). The collected data encompass basic information, family composition, physical and mental health status, healthcare coverage, employment and pension, financial resources and laboratory examinations. CHARLS commenced in 2011, with subsequent follow-ups conducted in 2013, 2015, 2018, and 2020. The Ethical Review Committee of Peking University granted approval for the CHARLS study (IRB00001052–11015). All participants obtained informed consent at the beginning of their study.

**Figure 1:**
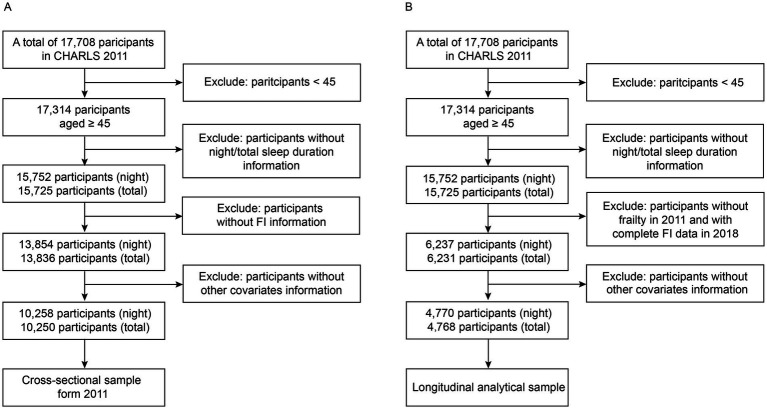
Flowchart of participant selection in the cross-sectional study **(A)** and the longitudinal study **(B)**.

The current investigation retrospectively analyzed data from the CHARLS 2011 and 2018. The baseline survey conducted in 2011 involved a total of 17,708 participants. The inclusion criteria for the cross-sectional study in 2011 were as follows: (1) complete information on individuals aged 45 years or older, including gender and other covariates; (2) complete data on night/total sleep duration; (3) complete data on the frailty index (Figure 1). Individuals who did not meet all of the above criteria were excluded. The inclusion criteria for the longitudinal study between 2011 and 2018 were as follows: (1) individuals aged 45 years or older with complete information on covariates at baseline; (2) complete data on night/total sleep duration; (3) individuals without frailty in 2011; (4) individuals with complete data on frailty index in 2018 (Figure 1). Individuals who did not meet all of the above criteria were excluded. Multiple imputations by chained equations with 5 imputations were conducted to impute missing covariate information in the sensitivity analysis. Estimates were obtained by pooling the results. The cross-sectional study in 2011 ultimately included 10,258/10,250 participants (night/total sleep duration), while the longitudinal study between 2011 and 2018 included 4,770/4,768 participants (night/total sleep duration).

### Sleep duration

The night sleep duration was evaluated through the following question: “During the past month, how many hours of actual sleep did you get at night (average hours for one night)?” The duration of nap time was evaluated using the following inquiry: “During the past month, how long did you take a nap after lunch?” We determined total sleep duration by adding together the duration of night sleep and nap time. Night sleep and total sleep were divided into three categories: short sleep duration (<6 h), normal sleep duration (6-9 h), and long sleep duration (≥9 h).

### Assessment of frailty index and frailty

The FI was developed by Rockwood et al. and is used for assessing frailty by quantifying the accumulation of age-related health deficits. The index can be customized and developed as needed by adhering to the health deficit selection principle (6). It is recommended to include a minimum of 30 health deficit items. The FI in this study is created from 33 terms, encompassing 14 chronic diseases, 5 disabilities, 12 limitations in activities of daily living (ADLs) and instrumental activities of daily living (IADLs), cognitive function and depression (Supplementary Table S1). The selection process is based on the established standard procedure and previous literature (8, 19–21). Chronic diseases identified in CHARLS include hypertension, diabetes, dyslipidemia, cancer, chronic lung disease, heart disease, stroke, psychiatric disorders, memory-related diseases, rheumatism, liver disease, kidney disease, digestive diseases, and asthma (22). Disabilities include physical disabilities, brain damage/mental retardation, vision problem, hearing problem and speech impediment (21, 22). Functional limitations are evaluated based on six questions for ADLs and six for IADLs (19). The cognitive function assessment encompasses an orientation test, a picture drawing test, a calculation test, and evaluations of both immediate and delayed memory capabilities, totaling 21 questions (21). The final cognitive score is a continuous variable obtained by dividing the total score by 21, with higher scores indicating worse cognitive function. Remaining terms were also assigned values from 0 to 1, with 0 indicating no health deficit and 1 indicating a health deficit. FI was calculated by dividing the total health deficit scores by 33 and multiplying by 100, with a range between 0 and 100. Higher scores suggest a higher level of frailty. As indicated in prior research, participants were categorized into physically robust (FI ≤ 10), pre-frailty (10 < FI < 25), and frailty (FI ≥ 25) groups (19, 23).

### Covariates

The analysis was adjusted for demographic characteristics, health behaviors, and anthropometric measurements. The covariates used in this study included age, sex, education level, marital status, residence, yearly expenditure, BMI, waist circumference, smoking status, and drinking status. Age was divided into two groups: 45 to 60 years old and 60 years old or older. Education level was classified as illiterate, middle school or below (home school, primary school and middle school) and high school or above (high school, vocational school, college/associate degree, bachelor’s degree, master’s degree and doctoral degree). We classified the marital status as either married or any other status, including unmarried, separated, divorced, or widowed. BMI was classified into three categories: underweight (BMI < 18.5 kg/m^2^), normal weight (18.5 kg/m^2^ ≤ BMI < 24 kg/m^2^), and overweight or obesity (BMI ≥ 24 kg/m^2^). We determined the smoking status based on the individual’s smoking history. The drinking status of individuals was assessed based on their alcohol consumption within the previous 12 months.

### Statistical analysis

Baseline characteristics were summarized according to sleep duration. The categorical variables were presented as n (%) and were compared using the chi-squared test. The continuous variables were presented as the mean ± standard deviation (SD) and were compared using one-way ANOVA.

In both cross-sectional and longitudinal study, linear regression and RCS analysis were used to assess the association between sleep duration and FI, while multinomial logistic regression analysis was used to examine the relationship between sleep duration and the risk of frailty. Multinomial logistic regression belongs to the generalized linear model family. This model uses a link function to mathematically connect the linear combination of input variables and their coefficients (linear predictor) to the expected probability of each categorical outcome. This transforms the typical S-shaped curve of logistic models into a linear relationship for analysis. Restricted cubic spline is a way to identify non-linear relationship, which essentially is a piecewise cubic polynomial (24). Each spline connects smoothly at each knot. To improve model fitting and smoothness while reduce overfitting, four knots were set at the 5th, 25th, 75th, and 95th percentiles of latitude, as recommended by Harrell (25). The selection of covariates was based on existing literature, and three models were used in the analysis (22). Model 1 is a crude model without any adjustments; Model 2 included adjustments for age, sex, educational level, marital status, residence and yearly expenditure; Model 3 adjusted for age, gender, educational level, marital status, residence, yearly expenditure, BMI, waist circumference, smoking status, and drinking status. Sensitivity analysis was conducted on the dataset after the imputation of incomplete covariate information. Subgroup analysis and interaction analysis were performed to investigate potential effect modifications of covariates on the association between sleep duration and frailty. Some subgroups were not statistically significant due to insufficient sample size such as “High school or above,” “Unmarried” and “Urban residence” subgroups in the longitudinal study, which comprised fewer than 1,000 individuals. All statistical analysis was performed by R software (Version 4.3.2). All *p* values were two-sided, and the statistical significance was defined as *p* < 0.05.

## Results

### Participant characteristics

Table 1 presents the baseline characteristics of the cross-sectional study population grouped by different night sleep durations. A total of 10,258 participants were included in the cross-sectional study of night sleep duration and FI/frailty. This sample consisted of 5,738 middle-aged adults aged between 45 and 60 years and 4,520 older adult adults aged 60 years and above (mean [SD] age: 59.1 [9.3] years). In the sample, 51.3% of the participants were female, 62.2% had middle school or below education, 88.3% were married, 80.5% resided in rural areas, 52.5% had normal weight, 59.1% were non-smokers, and 67.0% never consumed alcohol. The prevalence of frailty in the overall population is 7.9%.

**Table 1:** Baseline characteristics of all participants in night sleep duration analysis in 2011 (*n* = 10,258).

	Level	Overall	Short sleep duration	Normal sleep duration	Long sleep duration	*p*
n		10,258	2,999	6,456	803	
Sex (%)	Male	4,999 (48.7)	1,312 (43.7)	3,314 (51.3)	373 (46.5)	<0.001
	Female	5,259 (51.3)	1,687 (56.3)	3,142 (48.7)	430 (53.5)	
Age (mean (SD))		59.1 (9.3)	60.9 (9.4)	58.2 (9.1)	59.8 (10.1)	<0.001
(%)	<60	5,738 (55.9)	1,427 (47.6)	3,872 (60.0)	439 (54.7)	<0.001
	≥60	4,520 (44.1)	1,572 (52.4)	2,584 (40.0)	364 (45.3)	
Education level (%)	Illiterate	2,706 (26.4)	997 (33.2)	1,440 (22.3)	269 (33.5)	<0.001
	Middle school or below	6,376 (62.2)	1,810 (60.4)	4,096 (63.4)	470 (58.5)	
	High school or above	1,176 (11.5)	192 (6.4)	920 (14.3)	64 (8.0)	
Marital status (%)	Married	9,053 (88.3)	2,527 (84.3)	5,838 (90.4)	688 (85.7)	<0.001
	Others	1,205 (11.7)	472 (15.7)	618 (9.6)	115 (14.3)	
Residence status (%)	Rural	8,255 (80.5)	2,536 (84.6)	5,019 (77.7)	700 (87.2)	<0.001
	Urban	2,003 (19.5)	463 (15.4)	1,437 (22.3)	103 (12.8)	
Yearly expenditure (%)	Low	3,388 (33.0)	1,058 (35.3)	2,021 (31.3)	309 (38.5)	<0.001
	Medium	3,567 (34.8)	1,066 (35.5)	2,228 (34.5)	273 (34.0)	
	High	3,303 (32.2)	875 (29.2)	2,207 (34.2)	221 (27.5)	
BMI (%)	Underweight	703 (6.9)	280 (9.3)	351 (5.4)	72 (9.0)	<0.001
	Normal weight	5,389 (52.5)	1,647 (54.9)	3,305 (51.2)	437 (54.4)	
	Overweight or obesity	4,166 (40.6)	1,072 (35.7)	2,800 (43.4)	294 (36.6)	
Waist (mean (SD))		85.3 (10.3)	84.3 (10.3)	85.8 (10.3)	84.7 (10.1)	<0.001
Smoking (%)	Yes	4,197 (40.9)	1,158 (38.6)	2,734 (42.3)	305 (38.0)	<0.001
	No	6,061 (59.1)	1,841 (61.4)	3,722 (57.7)	498 (62.0)	
Drinking (%)	Yes	3,383 (33.0)	893 (29.8)	2,251 (34.9)	239 (29.8)	<0.001
	No	6,875 (67.0)	2,106 (70.2)	4,205 (65.1)	564 (70.2)	
FI (mean (SD))		10.9 (8.8)	14.1 (9.7)	9.5 (7.9)	10.9 (9.2)	<0.001
Frailty (%)	Frailty	814 (7.9)	405 (13.5)	340 (5.3)	69 (8.6)	<0.001
	Pre-frailty	3,837 (37.4)	1,411 (47.0)	2,141 (33.2)	285 (35.5)	
	Robust	5,607 (54.7)	1,183 (39.4)	3,975 (61.6)	449 (55.9)	

The cross-sectional study comprised 2,999 participants with short sleep duration, 6,456 with normal sleep duration, and 803 with long sleep duration. Participants in the short night sleep group were more likely to be female, older, less educated, single, with lower annual expenses, underweight, with smaller waists, less likely to smoke or drink and more prone to pre-frailty and frailty compared to the normal group. The characteristics of the participants in the longitudinal study of night sleep were approximately similar (Table 2). This is also manifested in cross-sectional and longitudinal studies of total sleep (Supplementary Tables S2, S3).

**Table 2:** Baseline characteristics of all participants in the longitudinal analysis of night sleep duration from 2011 to 2018 (*n* = 4,770).

	Level	Overall	Short sleep duration	Normal sleep duration	Long sleep duration	*p*
n		4,770	1,181	3,251	338	
Sex (%)	Male	2,295 (48.1)	522 (44.2)	1,611 (49.6)	162 (47.9)	0.007
	Female	2,475 (51.9)	659 (55.8)	1,640 (50.4)	176 (52.1)	
Age mean (SD)		56.1 (7.6)	57.5 (7.7)	55.6 (7.5)	55.5 (7.8)	<0.001
(%)	<60	3,294 (69.1)	747 (63.3)	2,311 (71.1)	236 (69.8)	<0.001
	≥60	1,476 (30.9)	434 (36.7)	940 (28.9)	102 (30.2)	
Education level (%)	Illiterate	925 (19.4)	291 (24.6)	553 (17.0)	81 (24.0)	<0.001
	Middle school or below	3,154 (66.1)	786 (66.6)	2,149 (66.1)	219 (64.8)	
	High school or above	691 (14.5)	104 (8.8)	549 (16.9)	38 (11.2)	
Marital status (%)	Married	4,426 (92.8)	1,076 (91.1)	3,035 (93.4)	315 (93.2)	0.036
	Others	344 (7.2)	105 (8.9)	216 (6.6)	23 (6.8)	
Residence status (%)	Rural	3,845 (80.6)	1,009 (85.4)	2,549 (78.4)	287 (84.9)	<0.001
	Urban	925 (19.4)	172 (14.6)	702 (21.6)	51 (15.1)	
Yearly expenditure (%)	Low	1,351 (28.3)	352 (29.8)	886 (27.3)	113 (33.4)	0.01
	Medium	1,745 (36.6)	440 (37.3)	1,177 (36.2)	128 (37.9)	
	High	1,674 (35.1)	389 (32.9)	1,188 (36.5)	97 (28.7)	
BMI (%)	Underweight	230 (4.8)	92 (7.8)	121 (3.7)	17 (5.0)	<0.001
	Normal weight	2,462 (51.6)	639 (54.1)	1,649 (50.7)	174 (51.5)	
	Overweight or obesity	2,078 (43.6)	450 (38.1)	1,481 (45.6)	147 (43.5)	
Waist (mean (SD))		85.5 (10.0)	84.5 (10.0)	85.9 (10.0)	85.6 (9.5)	<0.001
Smoking (%)	Yes	1,874 (39.3)	447 (37.8)	1,299 (40.0)	128 (37.9)	0.383
	No	2,896 (60.7)	734 (62.2)	1,952 (60.0)	210 (62.1)	
Drinking (%)	Yes	1,688 (35.4)	386 (32.7)	1,194 (36.7)	108 (32.0)	0.018
	No	3,082 (64.6)	795 (67.3)	2,057 (63.3)	230 (68.0)	
FI (mean (SD))		12.4 (8.9)	14.6 (9.4)	11.6 (8.6)	11.6 (8.4)	<0.001
Frailty (%)	Frailty	464 (9.7)	166 (14.1)	269 (8.3)	29 (8.6)	<0.001
	Pre-frailty	2,054 (43.1)	576 (48.8)	1,351 (41.6)	127 (37.6)	
	Robust	2,252 (47.2)	439 (37.2)	1,631 (50.2)	182 (53.8)	

### Cross-sectional associations between sleep duration and FI

In the cross-sectional study, a negative correlation was identified between both night and total sleep duration and FI in the crude model (Model 1, night sleep: *β* = −1.04, *p* < 0.001, total sleep: *β* = −0.83, *p* < 0.001) (Table 3). After adjusting for demographic characteristics (Model 2), and further for health habits and anthropometric measurements (Model 3), the association remained significant (Model 2, night sleep: *β* = −0.81, *p* < 0.001, total sleep: *β* = −0.63, *p* < 0.001; Model 3, night sleep: *β* = −0.83, *p* < 0.001, total sleep: *β* = −0.66, *p* < 0.001). After categorizing sleep duration into three groups, short night or total sleep duration increased FI in unadjusted and adjusted models compared to normal sleep duration (Table 3). Long sleep duration initially increased FI in models of night sleep but not total sleep. The findings suggest that short night and total sleep duration are associated with a higher FI.

**Table 3:** Linear and logistic regression analysis examining the relationship between sleep duration and frailty index or frailty in 2011 (*n* = 10,258/10,250).

	Model 1		Model 2		Model 3	
	FI	Frailty	FI	Frailty	FI	Frailty
Night sleep duration	−1.04 (0.05)***	0.98 [0.98, 0.98]***	−0.81 (0.04)***	0.99 [0.98, 0.99]***	−0.83 (0.04)***	0.99 [0.98, 0.99]***
Normal	Ref	Ref	Ref	Ref	Ref	Ref
Short	4.65 (0.19)***	1.09 [1.07, 1.10]***	3.50 (0.18)***	1.06 [1.05, 1.07]***	3.59 (0.18)***	1.06 [1.05, 1.08]***
Long	1.44 (0.32) ***	1.03 [1.01, 1.05]***	0.63 (0.30)*	1.02 [1.00, 1.04]	0.67 (0.30)*	1.02 [1.00, 1.04]
p for trend	*p*<0.001	*p* <0.001	*p* <0.001	*p* <0.001	*p* <0.001	*p* <0.001
Total sleep duration	−0.83 (0.04)***	0.99 [0.98, 0.99]***	−0.63 (0.04)***	0.99 [0.99, 0.99]***	−0.66 (0.04)***	0.99 [0.99, 0.99]***
Normal	Ref	Ref	Ref	Ref	Ref	Ref
Short	4.94 (0.21)***	1.10 [1.08, 1.11]***	3.66 (0.20)***	1.07 [1.06, 1.08]***	3.74 (0.20)***	1.07 [1.06, 1.08]***
Long	0.42 (0.22)	1.02 [1.00, 1.03]*	0.10 (0.20)	1.01 [1.00, 1.02]	0.01 (0.20)	1.01 [0.99, 1.02]
p for trend	*p*<0.001	*p*<0.001	*p*<0.001	*p*<0.001	*p*<0.001	*p*<0.001

Model 1 was crude model. Model 2 was adjusted for age, gender, education level, location and marital status. Model 3 was adjusted for age, gender, education level, location, marital status, BMI, waist, smoking status and drinking status. The *p*-value for the trend was computed over a range spanning from short to long sleep duration. n = night sleep duration number or (/) total sleep duration number. The data are presented as β (SE) or OR [95% CI]. FI, frailty index. *p < 0.05, **p < 0.01, ***p < 0.001.

### Cross-sectional associations between sleep duration and frailty

In the cross-sectional study, the risk of frailty was negatively associated with both night and total sleep duration (night sleep: OR [95 CI%], 0.98 [0.98, 0.98], *p* < 0.001; total sleep: 0.99 [0.98, 0.99], *p* < 0.001) (Table 3). The association retained statistical significance after adjusting for confounding variables (Model 2, night sleep: 0.99 [0.98, 0.99], *p* < 0.001, total sleep: 0.99 [0.99–0.99], *p* < 0.001; Model 3, night sleep: 0.99 [0.98, 0.99], *p* < 0.001, total sleep: 0.99 [0.99, 0.99], *p* < 0.001). After categorizing sleep duration into three groups, short sleep duration is associated with an increased risk of frailty compared to normal sleep duration in three models. Long sleep duration only increased the risk of frailty in the unadjusted model and this significance dissipated following adjustment. This suggests that short night sleep duration, as well as short total sleep duration, is associated with an increased risk of frailty.

### Longitudinal associations between sleep duration and FI

In the longitudinal study spanning from 2011 to 2018, FI exhibited a negative correlation with both night and total sleep duration in the crude model (Model 1, night sleep: *β* = −0.75, *p* < 0.001, total sleep: *β* = −0.62, *p* < 0.001) (Table 4). After adjusting for demographic characteristics, as well as health habits and anthropometric measurements, the association continued to exhibit statistical significance (Model 2, night sleep: *β* = −0.57, *p* < 0.001, total sleep: *β* = −0.43, *p* < 0.001; Model 3, night sleep: *β* = −0.59, *p* < 0.001, total sleep: *β* = −0.47, *p* < 0.001). The FI was significantly higher in short sleep group compared to normal sleep group (Table 4). The association still existed in the sensitivity analysis (Supplementary Table S4). This result is consistent with cross-sectional study indicating that shorter sleep duration may lead to increased FI.

**Table 4:** Linear and logistic regression analysis examining the longitudinal relationship between sleep duration and frailty index or frailty from 2011 to 2018 (*n* = 4,770/4,768).

	Model 1		Model 2		Model 3	
	FI	Frailty	FI	Frailty	FI	Frailty
Night sleep duration	−0.75 (0.07)***	0.99 [0.98, 0.99]***	−0.57 (0.07)***	0.99 [0.99, 1.00]***	−0.59 (0.07)***	0.99 [0.99, 1.00]***
Normal	Ref	Ref	Ref	Ref	Ref	Ref
Short	3.00 (0.30)***	1.06 [1.04, 1.08]***	2.22 (0.29)***	1.04 [1.02, 1.06]***	2.34 (0.29)***	1.05 [1.03, 1.07]***
Long	−0.03 (0.50)	1.00 [0.97, 1.04]	−0.22 (0.48)	1.00 [0.97, 1.03]	−0.22 (0.48)	1.00 [0.97, 1.03]
p for trend	*p*<0.001	*p* <0.001	*p* <0.001	*p* <0.001	*p* <0.001	*p* <0.001
Total sleep duration	−0.62 (0.07)***	0.99 [0.99, 0.99]***	−0.43 (0.06)***	0.99 [0.99, 1.00]**	−0.47 (0.06)***	0.99 [0.99, 1.00]***
Normal	Ref	Ref	Ref	Ref	Ref	Ref
Short	2.91 (0.33)***	1.05 [1.03, 1.08]***	2.03 (0.32) ***	1.04 [1.01, 1.06]**	2.21 (0.32)***	1.04 [1.02, 1.06]***
Long	−0.53 (0.32)	0.99 [0.97, 1.01]	−0.36 (0.31)	0.99 [0.97, 1.01]	−0.43 (0.30)	0.99 [0.97, 1.01]
p for trend	*p*<0.001	*p*<0.001	*p*<0.001	*p*<0.01	*p*<0.001	*p*<0.001

Model 1 was crude model. Model 2 was adjusted for age, gender, education level, location and marital status. Model 3 was adjusted for age, gender, education level, location, marital status, BMI, waist, smoking status and drinking status. The *p*-value for the trend was computed over a range spanning from short to long sleep duration. n = night sleep duration number or (/) total sleep duration number. The data are presented as β (SE) or OR [95% CI]. FI, frailty index. * *p* < 0.05, ** *p* < 0.01, *** *p* < 0.001.

### Longitudinal associations between sleep duration and frailty

The longitudinal study also revealed a negative association between both night and total sleep duration and the risk of frailty across all three models (Model 1, night sleep: 0.99 [0.98, 0.99], *p* < 0.001, total sleep: 0.99 [0.99, 0.99], *p* < 0.001; Model 2, night sleep: 0.99 [0.99, 1.00], *p* < 0.001, total sleep: 0.99 [0.99, 1.00], *p* < 0.01; Model 3, night sleep: 0.99 [0.99, 1.00], *p* < 0.001, total sleep: 0.99 [0.99,1.00], *p* < 0.01) (Table 4). The incidence of frailty was increased in short sleep group compared to normal sleep duration. The relationship persisted in the sensitivity study (Supplementary Table S4). This suggests that short night sleep duration, as well as short total sleep duration, may increase the risk of frailty.

### Non-linear relationship between sleep duration and frailty

**Figure 2:**
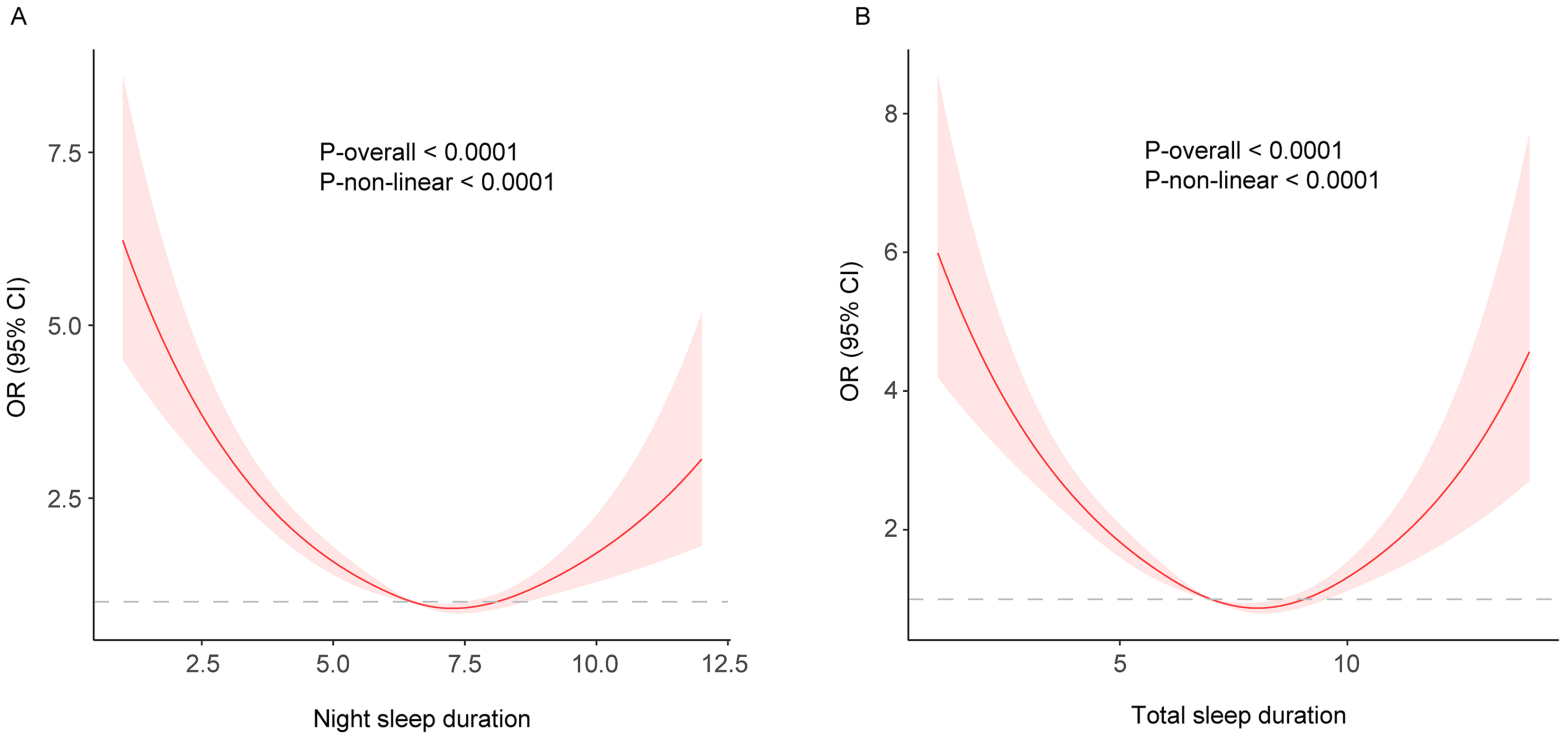
**(A)** night; **(B)** total. Restricted cubic spline of the association between night/total sleep duration and the risk of frailty in the cross-sectional study. The model was adjusted for age, sex, education level, marital status, residence, yearly expenditure, BMI, waist circumference, smoking and drinking status.

**Figure 3:**
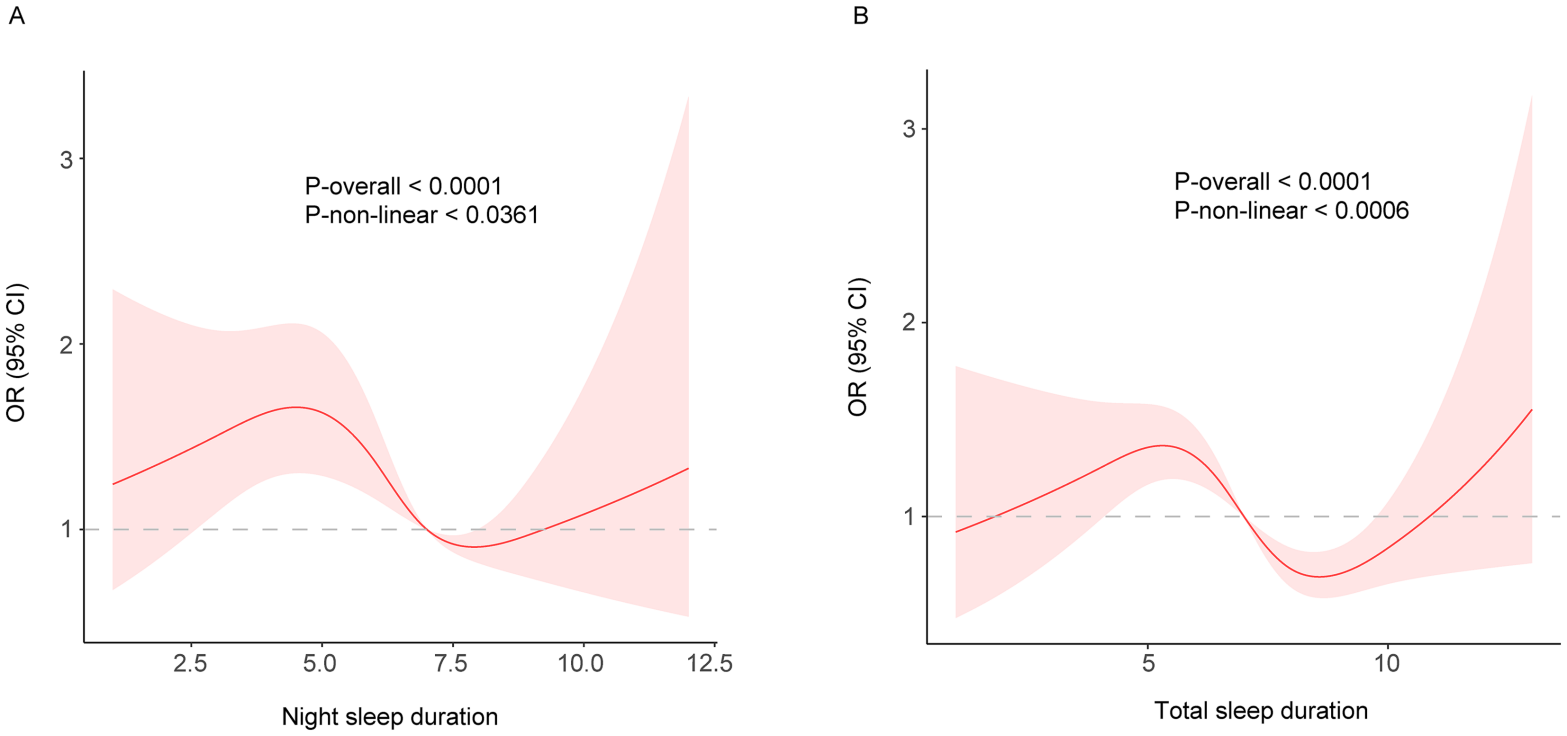
**(A)** night; **(B)** total. Restricted cubic spline of the association between night/total sleep duration and the risk of frailty in the longitudinal study. The model was adjusted for age, sex, education level, marital status, residence, yearly expenditure, BMI, waist circumference, smoking and drinking status.

While a linear relationship between sleep duration and FI was revealed both in the cross-sectional and longitudinal analysis, a non-linear relationship was also detected (P-nonlinear <0.001). Restricted cubic spline regression showed a non-linear (U-shaped) relationship between both night and total sleep duration and the risk of frailty using the fully adjusted model (Figures 2, 3). The OR value of 1 is depicted by a dotted horizontal line, which intersects the curve at 6.5 h and 8.1 h for night sleep, and at 7.0 h and 9.0 h for total sleep. In the cross-sectional study, the risk of frailty decreases as sleep duration increases up to 7.2 h per night or 8.1 h per day, followed by a reversal trend beyond these thresholds (Figure 2). In the longitudinal study, the curve remained almost the same except the right half becoming insignificant (turning point: night, 7.9 h; total, 8.6 h) (Figure 3). This suggests that both short and long sleep duration may increase the risk of frailty.

The result of long sleep duration in restricted cubic spline regression is different from the insignificant result of the fully adjusted linear model. In comparing the linear and non-linear models, the AIC and BIC values suggest that the linear model performed better than the non-linear model. Therefore, our study tends to believe that long sleep duration is less likely to increase the risk of frailty. The discrepancy may be attributed to the relatively smaller sample size in the long sleep duration group, which complicates the interpretation of the results. Nonetheless, both results have supporting evidence. A study from China Kadoorie Biobank which conducted in 10 regions across China found that short sleep duration, but not long sleep duration, increased the risk of exacerbating frailty (16). This study included only a brief assessment of depression and did not incorporate a cognitive evaluation. A study in the Netherlands found that both short and long sleep durations were associated with an increased risk of physical frailty (9). However, the association with long sleep duration lost significance after further adjustment. This study also found that both short and long sleep duration were associated with psychological frailty but not cognitive frailty. This suggests that the significant result of long sleep duration in our RSC analysis may be attributed to the relatively comprehensive assessment of depression or cognitive function in FI.

### Stratified and interaction analysis

**Figure 4:**
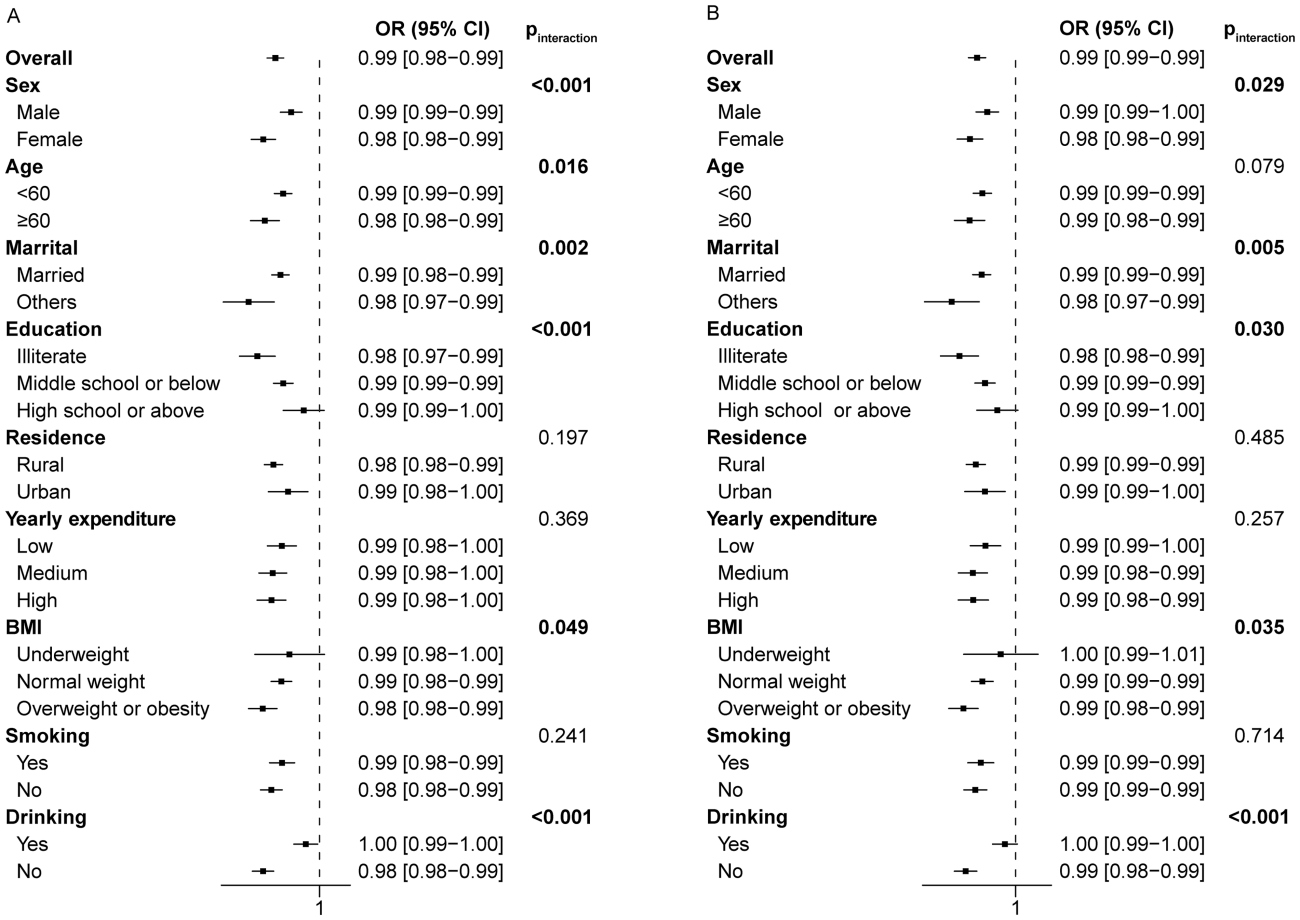
Forest plot of stratified analysis of the relationship between night/total sleep duration and the risk of frailty in the cross-sectional study. **(A)** The effect of night sleep duration on frailty in subgroups. **(B)** The effect of total sleep duration on frailty in subgroups.

To investigate whether sleep duration has varying effects on the risk of frailty among different subgroups, we performed a stratified analysis. The associations were robust in subgroup analysis in terms of age, gender, marital status, education level (excluding high school or above), residence, yearly expenditure, BMI (excluding underweight individuals), smoking and drinking status in the cross-sectional study of night sleep duration (Figure 4A). The insignificance observed in specific subgroups may be attributed to the relatively small sample sizes within these subgroups. Night sleep duration seems to have a greater impact on frailty in specific populations, such as females, individuals aged 60 and above, single individuals, those with limited literacy, and nondrinkers (Figure 4A). Likewise, significant interaction effects were also observed within subgroups of gender, marital status, education level, BMI, and drinking status, but not age in the cross-sectional analysis of total sleep duration (Figure 4B).

**Figure 5:**
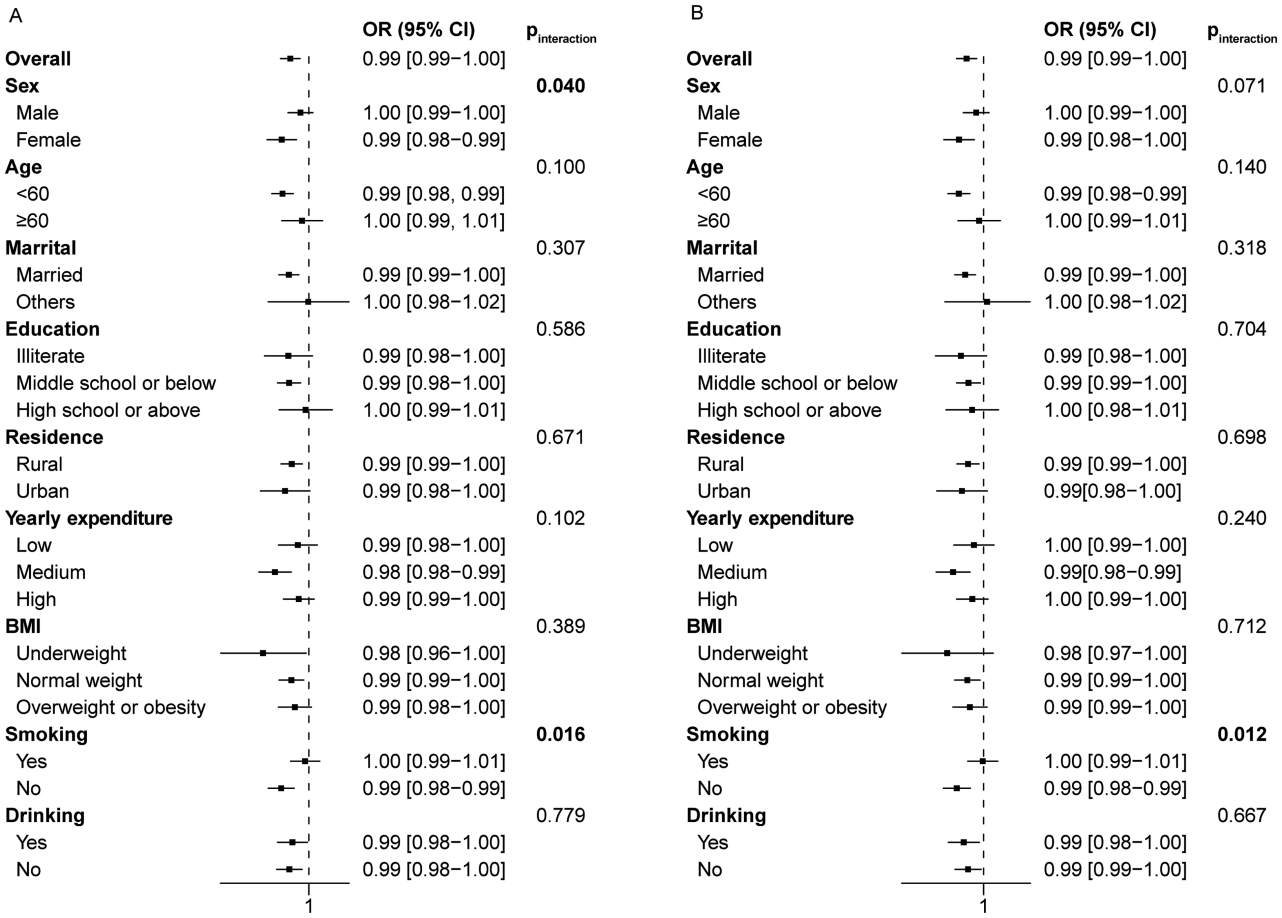
Forest plot of stratified analysis of the relationship between night/total sleep duration and the risk of frailty in the longitudinal study. **(A)** The effect of night sleep duration on frailty in subgroups. **(B)** The effect of total sleep duration on frailty in subgroups.

The longitudinal stratification analysis of night sleep duration showed consistent results with the main findings. However, for certain subgroups such as males, individuals aged 60 and above, illiterate, with a high school education or above, single, living in urban areas, with low or high annual expenses, underweight, overweight or obese, and smokers, the observed relationship was not statistically significant (Figure 5A). This may arise from the limited sample size in these subgroups as a result of lost follow-up. As a consequence, no significant interactions were observed among subgroups based on age, marital status, education level, residence, yearly expenditure, BMI and drinking status (p for interaction >0.05). However, a significant interaction was noted for smoking status. The analysis of total sleep duration yielded similar results (Figure 5B).

## Discussion

The average sleep duration has declined over the past few decades. This issue is particularly prevalent among the older adult population, primarily due to health issues, medication, and life transitions such as retirement, bereavement, and reduced social engagements (26, 27). While several studies have investigated the relationship between sleep duration and frailty in China, a consistent and comprehensive national conclusion is still lacking (5, 15–17). The discrepancy may be ascribed to the use of different assessment tools for frailty and different compositions of FI. Most of these studies conducted in China assessed frailty using the Fried phenotype, while one used FI but did not meet the recommended threshold of 30 deficits and did not include cognitive assessment (16). Our study covers a broader geographical area within China and includes a thorough evaluation of depression and cognitive function, thereby improving its overall comprehensiveness and reliability. (7). Although the association of short sleep duration with increased frailty has been identified in older adult Mexican women, this correlation is not observed in older adult American men or in both genders in Korea (15). In addition to the different frailty assessment tools used, the various covariant may also contribute to the discrepancies. Furthermore, the regional variations in frailty prevalence also suggest the existence of unidentified factors that may influence frailty (15, 28). This study observed an association between short sleep duration and an increased risk of frailty, while the relationship between long sleep duration and frailty remains inconclusive. On one hand, although the *p*-value for trend is significant and the linear model performed better than the non-linear model, long sleep duration does not exhibit significance in the linear model, especially after adjustment. On the other hand, long sleep duration lost significance in the non-linear model of longitudinal study. This is indicated by the 95% CI for the right half of the curve crossing the zero line. Our finding was supported by several Mendelian randomization studies using genetic data, which demonstrates a negative correlation between the sleep duration and FI (3, 29).

Short sleep duration is associated with many aspects of frailty. Chronic sleep loss is associated with a wide range of adverse health effects, including cardiovascular disease, hypertension, diabetes, stroke, cognitive deficits and depression (3, 30). All of these are constitutions of FI. Insufficient sleep also elevates the risk of developing sarcopenia. Sarcopenia reduces muscle mass and function, contributing to the functional limitations of frailty (3).

A potential explanation for this correlation could be that inadequate sleep duration is associated with increased inflammation markers and hormonal/metabolic disturbances. A NHANES study has demonstrated the mediating role of inflammation in the association between sleep quality and frailty. Sleep deprivation by reducing sleep duration from 8 to 6 h per night for 1 week resulted in elevated levels of IL-6 and TNF-*α* in a group of healthy young people (31). Besides, a systematic review and meta-analysis demonstrated a correlation between short sleep duration and elevated levels of C-reactive protein (CRP) (32). The elevated levels of these inflammatory molecules may aggravate the preexisting low-grade, chronic, systemic inflammatory state in older adult individuals, particularly those who are frail. In fact, elevated levels of IL-6 and TNF-α have been observed in the circulation of older adults, with frail individuals showing even higher levels after adjusting for age (7, 33, 34). A recent study reveals that CRP not only correlates with higher FI values but also increases the risk of frailty within an 8-year period (35). Elevated levels of inflammatory factors can have a direct or indirect impact on frailty. Elevated levels of IL-6 are linked to a reduction in muscle mass and strength, and can serve as a predictive factor for the development of physical disability in a longitudinal study (33). Furthermore, elevated IL-6 levels are associated with many age-related conditions, like atherosclerosis, arthritis, dementia and functional decline, all of which are components of FI (33, 36). In addition, a cross-sectional study has shown a correlation between sleep duration and pro-oxidant/antioxidant balance (37). The imbalance between reactive oxygen species and antioxidant capacity is a characteristic feature of the aging process and frailty (38, 39).

Decreased sleep duration can lead to negative impacts on hormonal and metabolic processes. Disturbed sleep may reduce growth hormone, insulin-like growth factor-1, and sex hormone secretion, which in turn enhance muscle proteolysis, thus leading to sarcopenia and frailty. The decrease in slow-wave sleep levels is primarily responsible for the decline in growth hormone secretion with age. Short sleep duration also decreases testosterone levels (40). The decrease in growth hormone and testosterone levels leads to reduced nitrogen retention and protein synthesis in muscles, resulting in a decline in muscle mass and function. In terms of catabolism, sleep restriction is linked to increased hypothalamic–pituitary–adrenal activity, leading to elevated cortisol levels. Cortisol is involved in muscle catabolism in individuals affected by aging, sarcopenia, and Cushing syndrome (26). From a metabolic perspective, reduction in sleep duration promotes proteolysis, leading to a decrease in muscle mass rather than adipose tissue loss (26). Insufficient sleep is associated with compromised insulin sensitivity and glucose metabolism (41). Insulin resistance is commonly observed in the older adult and is considered a contributing factor to sarcopenia (42). This is because insulin stimulates muscle anabolism and suppresses proteolysis. Impaired glucose metabolism enables protein to be used as an energy source in muscle tissue. In addition, the aforementioned pro-inflammatory cytokines can also induce proteolysis (3). Studies have shown the inverse relationship between circulating IL-6 levels and insulin-like growth factor 1 (IGF-1), an anabolic hormone secreted in response to growth hormone, in frail older adults instead of non-frail controls. Further investigation is necessary to elucidate the specific role of sleep in the progression of frailty.

The impact of sleep duration on frailty varies in subgroup analysis based on sex, marital status, education level, BMI, and drinking habits in the cross-sectional study. The insignificant interaction effect in the longitudinal study may be attributed to the inadequate sample size in subgroups. Previous studies have found a significant association between inadequate sleep and frailty in women but not in men (17, 43). Despite women’s longer life expectancy than men’s, there is a widely acknowledged higher prevalence of frailty in women (7). Previous research suggests that females have higher levels of inflammatory factors such as IL-6 and CRP, potentially explaining their increased vulnerability to frailty and the predisposing factors. Poor sleep has been associated with elevated CRP levels in women but not in men (17). There are also variations in sleep characteristics between men and women, with women generally exhibiting longer total sleep time and deeper sleep compared to men (43). This suggests that women’s longer biological needs may make a shorter sleep duration more significant for them. There are additional variables that may influence the impact of sleep duration on frailty, including marital status and education level. The variation across different marital statuses may be attributed to the correlation between high levels of loneliness and an elevated risk of frailty (44). The same scenario applies to lower education level (45).

The present study exhibits several strengths. Firstly, the CHARLS dataset used in the current study is a nationally representative survey with a substantial sample size of middle-aged and older adult adults in China. Consequently, it circumvents regional differences, ensuring that the conclusion is nationally representative. The inclusion of middle-aged individuals enhanced the study’s capacity to concentrate on frailty prevention. Secondly, this study investigated both the cross-sectional and longitudinal relationships between sleep duration and frailty, which facilitates the potential inference of a causal relationship. The total sleep duration result is consistent with night sleep duration, thereby strengthening the inference. Nevertheless, given the observational design of this study, further research, including intervention studies, is required to establish a causal relationship. Thirdly, this study adequately accounted for covariates, including variables previously linked to frailty prevalence, such as gender, age, education level and yearly expenditure (46). The study also has some limitations. Firstly, the lack of objective sleep measurements and reliance on self-reports may introduce recall bias, especially in older participants with cognitive decline. Previous studies have indicated that individuals tend to overestimate their sleep duration, often equating it with the total time spent in bed (47). However, given the strong correlation between bedtime and actual sleep duration, it is likely that self-reported long sleep duration is reflective of extended physiological sleep (10). Further research is needed to objectively measure sleep duration using polysomnography or actigraphy in order to reduce potential bias from inaccurate self-reporting. Secondly, while several important confounders have been considered, it is possible that residual confounding factors, such as insomnia, obstructive sleep apnea, restless legs syndrome or other psychiatric symptoms, may still exist. An observational study conducted in a city of China found that poor sleep quality is associated with frailty and pre-frailty in those aged 70 years and older using phenotype criteria (5). The similar finding was also reported in a study in Mexico (13). In another study which used multidimensional frailty criteria, a positive correlation was observed between insomnia and both cognitive frailty and psychological frailty. Nevertheless, the relationship between insomnia and physical frailty or total frailty remains unclear in this study (48). An American study found that obstructive sleep apnea is associated with an increased risk of frailty in men, but this was observed only in women in Mexico (13, 49). In addition to depression, frail individuals showed significantly higher levels of anxiety and stress than robust individuals (50). Another study indicated that only those with stress symptoms, but not anxiety, show a faster decline in physical function (51). More longitudinal studies and intervention studies may be required to adequately address this issue.

## Conclusion

This study provides evidence supporting the relationship between short sleep duration and frailty. The findings help identify people who are vulnerable to frailty. More research that uses objective sleep measurements and includes a wider range of diverse populations is necessary to draw a definitive conclusion.

Based on this finding, it is recommended that the government enhance public awareness and educational initiatives for middle-aged and older adult individuals to promote sufficient sleep. Family members of this population or the staff at the nursing home should pay more attention to their sleep duration when taking care of them. It is advisable for nursing homes to establish a standardized sleep schedule. Clinicians should consider the sleep duration of middle-aged and older adult patients during the treatment of various diseases and should intervene with pharmacological agents or other appropriate measures when necessary. These interventions are expected to decrease the prevalence of frailty in the future.

## Data Availability

Publicly available datasets were analyzed in this study. This data can be found at: https://charls.pku.edu.cn/.

## Footnotes

^1^ https://charls.pku.edu.cn/
