# Supplementary material for: The relationship between sleep duration and frailty: findings from the China Health and Retirement Longitudinal Study
Source: Front Public Health. 2024 Dec 23;12:1493533. doi: 10.3389/fpubh.2024.1493533 (PMC11701061; doi:10.3389/fpubh.2024.1493533)
Supplement: Supplementary file 1 [file Data_Sheet_1.docx]

**Supplementary Table 1. List of 33 health deficits items included in the frailty index**

|  | Item | Value |
| --- | --- | --- |
| Chronic diseases ^a^ | Hypertension | Yes=1, no=0 |
|  | Diabetes or high blood sugar | Yes=1, no=0 |
|  | Cancer or malignant tumor | Yes=1, no=0 |
|  | Chronic lung disease | Yes=1, no=0 |
|  | Heart attack, coronary heart disease, angina, congestive heart failure or other heart problems | Yes=1, no=0 |
|  | Stroke | Yes=1, no=0 |
|  | Emotional, nervous, psychiatric problems | Yes=1, no=0 |
|  | Memory-related disease ^b^ | Yes=1, no=0 |
|  | Arthritis or rheumatism | Yes=1, no=0 |
|  | Dyslipidemia | Yes=1, no=0 |
|  | Liver disease | Yes=1, no=0 |
|  | Kidney disease | Yes=1, no=0 |
|  | Stomach or other digestive disease | Yes=1, no=0 |
|  | Asthma | Yes=1, no=0 |
| Disability ^c^ | Physical disabilities | Yes=1, no=0 |
|  | Brain damage/mental retardation | Yes=1, no=0 |
|  | Vision problem | Yes=1, no=0 |
|  | Hearing problem | Yes=1, no=0 |
|  | Speech impediment | Yes=1, no=0 |
| Functional limitation ^d^ | Doing household chores | No=0; a little =0.33; need help = 0.67; Yes=1 |
|  | Preparing hot meals | No=0; a little =0.33; need help = 0.67; Yes=1 |
|  | Managing assets | No=0; a little =0.33; need help = 0.67; Yes=1 |
|  | Taking medications | No=0; a little =0.33; need help = 0.67; Yes=1 |
|  | Shopping because of health and memory problems | No=0; a little =0.33; need help = 0.67; Yes=1 |
|  | Running or jogging About 1 kilometer | No=0; a little =0.33; need help = 0.67; Yes=1 |
|  | Getting up from a chair after sitting | No=0; a little =0.33; need help = 0.67; Yes=1 |
|  | Climbing several flights of stairs without rest | No=0; a little =0.33; need help = 0.67; Yes=1 |
|  | Stooping, kneeling, or crouching | No=0; a little =0.33; need help = 0.67; Yes=1 |
|  | Reaching or extending your arms above shoulder | No=0; a little =0.33; need help = 0.67; Yes=1 |
|  | Lifting or carrying weights over 10 jin (about 5 kilograms) | No=0; a little =0.33; need help = 0.67; Yes=1 |
|  | Picking up a small coin from a table | No=0; a little =0.33; need help = 0.67; Yes=1 |
| Cognitive function ^e^ | (picture drawing + word recall + orientation + calculation) / 21 | Continuous, ranging from 0 to 1 |
| Depression ^f^ | CESD-10 | CESD-10 >10 =1, ≤10 =0 |

^a^ Self-reported chronic disease, ascertained by participants’ answers to the question: “Have you been diagnosed with [diseases] by a doctor?”

^b^ Memory-related disease indicates Alzheimer’s disease or dementia, organic brain senility, or other serious memory impairment.

^c^ Self-reported disabilities, ascertained by participants’ answers to the question: “Do you have the following disabilities?”

^d^ Self-reported functional limitations, ascertained by participants’ answers to the questions “Do you have difficulty with…?”

^e^ The word recall is the average of words that are not recalled in the immediate and delayed word recall tasks, with a scale from 0 to 10. The orientation test consists of five questions related to the date, the month, and the year, the day of the week, the current season. Each incorrect response is scored as one point, with a range from 0 to 5. Calculation refers to the numbers of incorrect answers to following series questions: " What is the result of subtracting 7 from 100? Subtract 7 from the previous result, and repeat this process four more times.".

^f^ Depression is assessed using 10-item Centre for Epidemiologic Studies Depression Scale (CESD-10), with a total score ranging from 0 to 30. Higher scores indicate more severe depressive symptoms.

**Supplementary Table 2. Baseline characteristics of all participants in total sleep duration analysis in 2011 (n =10,250)**

|  | **Level** | **Overall** | **Short sleep duration (total)** | **Normal sleep duration (total)** | **Long sleep duration (total)** | **p** |
| --- | --- | --- | --- | --- | --- | --- |
| n |  | 10,250 | 2,383 | 5,658 | 2,209 |  |
| Sex (%) | Male | 4,996 (48.7) | 952 (39.9) | 2,863 (50.6) | 1,181 (53.5) | <0.001 |
|  | Female | 5,254 (51.3) | 1,431 (60.1) | 2,795 (49.4) | 1,028 (46.5) | |
| Age (mean (SD)) | | 59.1 (9.3) | 61.0 (9.5) | 58.2 (9.0) | 59.2 (9.8) | <0.001 |
| (%) | <60 | 5,735 (56.0) | 1,123 (47.1) | 3,383 (59.8) | 1,229 (55.6) | <0.001 |
|  | ≥60 | 4,515 (44.0) | 1,260 (52.9) | 2,275 (40.2) | 980 (44.4) | |
| Education level (%) | Illiterate | 2,702 (26.4) | 868 (36.4) | 1,259 (22.3) | 575 (26.0) | <0.001 |
|  | Middle school or below | 6,372 (62.2) | 1,381 (58.0) | 3,601 (63.6) | 1,390 (62.9) | |
|  | High school or above | 1,176 (11.5) | 134 (5.6) | 798 (14.1) | 244 (11.0) |  |
| Marital status (%) | Married | 9,046 (88.3) | 1,985 (83.3) | 5,103 (90.2) | 1,958 (88.6) | <0.001 |
|  | Others | 1,204 (11.7) | 398 (16.7) | 555 (9.8) | 251 (11.4) | |
| Residence status (%) | Rural | 8,250 (80.5) | 2,014 (84.5) | 4,398 (77.7) | 1,838 (83.2) | <0.001 |
|  | Urban | 2,000 (19.5) | 369 (15.5) | 1,260 (22.3) | 371 (16.8) | |
| Yearly expenditure (%) | Low | 3,385 (33.0) | 862 (36.2) | 1,727 (30.5) | 796 (36.0) | <0.001 |
|  | Medium | 3,564 (34.8) | 838 (35.2) | 1,971 (34.8) | 755 (34.2) |  |
|  | High | 3,301 (32.2) | 683 (28.7) | 1,960 (34.6) | 658 (29.8) |  |
| BMI (%) | Underweight | 702 (6.8) | 238 (10.0) | 323 (5.7) | 141 (6.4) | <0.001 |
|  | Normal weight | 5,387 (52.6) | 1,329 (55.8) | 2,932 (51.8) | 1,126 (51.0) | |
|  | Overweight or obesity | 4,161 (40.6) | 816 (34.2) | 2,403 (42.5) | 942 (42.6) | |
| Waist (mean (SD)) | | 85.3 (10.3) | 83.9 (10.1) | 85.5 (10.4) | 86.1 (10.3) | <0.001 |
| Smoking (%) | Yes | 4,194 (40.9) | 860 (36.1) | 2,371 (41.9) | 963 (43.6) | <0.001 |
|  | No | 6,056 (59.1) | 1,523 (63.9) | 3,287 (58.1) | 1,246 (56.4) | |
| Drinking (%) | Yes | 3,380 (33.0) | 641 (26.9) | 1,955 (34.6) | 784 (35.5) | <0.001 |
|  | No | 6,870 (67.0) | 1,742 (73.1) | 3,703 (65.4) | 1,425 (64.5) | |
| FI (mean (SD)) | | 10.9 (8.8) | 14.6 (9.9) | 9.7 (7.9) | 10.1 (8.7) | <0.001 |
| Frailty (%) | Frailty | 813 (7.9) | 349 (14.6) | 310 (5.5) | 154 (7.0) | <0.001 |
|  | Pre-frailty | 3,835 (37.4) | 1,143 (48.0) | 1,945 (34.4) | 747 (33.8) |  |
|  | Robust | 5,602 (54.7) | 891 (37.4) | 3,403 (60.1) | 1,308 (59.2) |  |

**Supplementary Table 3. Baseline characteristics of all participants in the longitudinal analysis of total sleep duration from 2011 to 2018 (n = 4,768).**

|  | **Level** | **Overall** | **Short sleep duration (total)** | **Normal sleep duration (total)** | **Long sleep duration (total)** | | **p** |
| --- | --- | --- | --- | --- | --- | --- | --- |
| n |  | 4,768 | 919 | 2,817 | 1,032 | |  |
| Sex (%) | Male | 2,293 (48.1) | 371 (40.4) | 1,376 (48.8) | 546 (52.9) | | <0.001 |
|  | Female | 2,475 (51.9) | 548 (59.6) | 1,441 (51.2) | 486 (47.1) |  | |
| Age (mean (SD)) | | 56.1 (7.6) | 57.6 (7.8) | 55.8 (7.5) | 55.4 (7.7) | | <0.001 |
| (%) | <60 | 3,293 (69.1) | 576 (62.7) | 1,985 (70.5) | 732 (70.9) | | <0.001 |
|  | ≥60 | 1,475 (30.9) | 343 (37.3) | 832 (29.5) | 300 (29.1) |  | |
| Education level (%) | Illiterate | 925 (19.4) | 247 (26.9) | 498 (17.7) | 180 (17.4) | | <0.001 |
|  | Middle school or below | 3,152 (66.1) | 600 (65.3) | 1,846 (65.5) | 706 (68.4) |  | |
|  | High school or above | 691 (14.5) | 72 (7.8) | 473 (16.8) | 146 (14.1) | |  |
| Marital status (%) | Married | 4,425 (92.8) | 835 (90.9) | 2,626 (93.2) | 964 (93.4) | | 0.039 |
|  | Others | 343 (7.2) | 84 (9.1) | 191 (6.8) | 68 (6.6) |  | |
| Residence status (%) | Rural | 3,844 (80.6) | 786 (85.5) | 2,205 (78.3) | 853 (82.7) | | <0.001 |
|  | Urban | 924 (19.4) | 133 (14.5) | 612 (21.7) | 179 (17.3) |  | |
| Yearly expenditure (%) | Low | 1,350 (28.3) | 283 (30.8) | 745 (26.4) | 322 (31.2) | | 0.006 |
|  | Medium | 1,745 (36.6) | 329 (35.8) | 1,036 (36.8) | 380 (36.8) | |  |
|  | High | 1,673 (35.1) | 307 (33.4) | 1,036 (36.8) | 330 (32.0) | |  |
| BMI (%) | Underweight | 229 (4.8) | 77 (8.4) | 107 (3.8) | 45 (4.4) | | <0.001 |
|  | Normal weight | 2,462 (51.6) | 508 (55.3) | 1,428 (50.7) | 526 (51.0) |  | |
|  | Overweight or obesity | 2,077 (43.6) | 334 (36.3) | 1,282 (45.5) | 461 (44.7) |  | |
| Waist (mean (SD)) | | 85.5 (10.0) | 84.0 (9.7) | 85.8 (10.1) | 86.1 (9.9) | | <0.001 |
| Smoking (%) | Yes | 1,872 (39.3) | 318 (34.6) | 1,116 (39.6) | 438 (42.4) | | 0.002 |
|  | No | 2,896 (60.7) | 601 (65.4) | 1,701 (60.4) | 594 (57.6) |  | |
| Drinking (%) | Yes | 1,687 (35.4) | 273 (29.7) | 1,029 (36.5) | 385 (37.3) | | <0.001 |
|  | No | 3,081 (64.6) | 646 (70.3) | 1,788 (63.5) | 647 (62.7) |  | |
| FI (mean (SD)) | | 12.4 (8.9) | 14.9 (9.3) | 11.9 (8.7) | 11.4 (8.6) | | <0.001 |
| Frailty (%) | Frailty | 464 (9.7) | 131 (14.3) | 252 (8.9) | 81 (7.8) | | <0.001 |
|  | Pre-frailty | 2,052 (43.0) | 463 (50.4) | 1,171 (41.6) | 418 (40.5) |  | |
|  | Robust | 2,252 (47.2) | 325 (35.4) | 1,394 (49.5) | 533 (51.6) |  | |

**Supplementary Table 4. Linear and logistic regression analysis examining the longitudinal relationship between sleep duration and frailty index or frailty from 2011 to 2018 for** **sensitivity analysis (n = 6,237/6,231).**

|  | Model 1 | | Model 2 | | Model 3 | |
| --- | --- | --- | --- | --- | --- | --- |
|  | **FI** | **Frailty** | **FI** | **Frailty** | **FI** | **Frailty** |
| Night sleep duration | -0.75 (0.07) *** | 0.87 [0.83, 0.91] *** | -0.59 (0.06) *** | 0.90 [0.86, 0.94] *** | -0.61 (0.06) *** | 0.89 [0.85, 0.94] *** |
| Normal | Ref | Ref | Ref | Ref | Ref | Ref |
| Short | 3.08 (0.26) *** | 1.83 [1.53, 2.19] *** | 2.39 (0.26) *** | 1.60 [1.33, 1.93] *** | 2.49 (0.25) *** | 1.66 [1.37, 2.00] *** |
| Long | -0.06 (0.44) | 0.99 [0.70, 1.41] | -0.23 (0.42) | 0.95 [0.66, 1.36] | -0.21 (0.42) | 0.97 [0.68, 1.39] |
| p for trend | p＜0.001 | p ＜0.001 | p ＜0.001 | p ＜0.001 | p ＜0.001 | p ＜0.001 |
| Total sleep duration | -0.64 (0.06) *** | 0.88 [0.84,0.92] *** | -0.48 (0.06) *** | 0.91 [0.88, 0.95] *** | -0.51 (0.06) *** | 0.91 [0.87, 0.95] *** |
| Normal | Ref | Ref | Ref | Ref | Ref | Ref |
| Short | 3.07 (0.30) *** | 1.77 [1.45, 2.16] *** | 2.25 (0.29) *** | 1.51 [1.23, 1.85] *** | 2.41 (0.29) *** | 1.58 [1.28, 1.94] *** |
| Long | -0.39 (0.28) | 0.91 [0.72, 1.14] | -0.31 (0.27) | 0.92 [0.73, 1.16] | -0.39 (0.27) | 0.91 [0.72, 1.15] |
| p for trend | p＜0.001 | p＜0.001 | p＜0.001 | p＜0.001 | p＜0.001 | p＜0.001 |

Model 1 was crude model. Model 2 was adjusted for age, gender, education level, location and marital status. Model 3 was adjusted for age, gender, education level, location, marital status, BMI, waist, smoking status and drinking status. n = night sleep duration number or (/) total sleep duration number. The data are presented as β (SE) or OR [95% CI]. FI, frailty index. * p<0.05, ** p<0.01, *** p<0.001.
